# Relationship between parent–adolescent communication and parent involvement in adolescent Type 1 diabetes management, parent/family wellbeing and glycaemic control

**DOI:** 10.1177/17423953231184423

**Published:** 2023-06-29

**Authors:** Ailbhe Benson, Caroline Rawdon, Ella Tuohy, Nuala Murphy, Ciara McDonnell, Veronica Swallow, Pamela Gallagher, Veronica Lambert

**Affiliations:** 1 School of Nursing, Psychotherapy and Community Health, Faculty of Science and Health, 8818 Dublin City University, Dublin, Ireland; 2 School of Psychology, Faculty of Science and Health, 8818 Dublin City University, Dublin, Ireland; 3 Diabetes and Endocrine Unit, Children's Health Ireland, Dublin, Ireland; 4 91105 Trinity Research in Childhood Centre, School of Medicine, University of Dublin, Trinity College Dublin, Dublin, Ireland; 5 College of Health, WellBeing and Life Sciences, 7314 Sheffield Hallam University, Sheffield, UK

**Keywords:** Chronic illness, diabetes mellitus, type 1, adolescence, communication, parents

## Abstract

**Objectives:**

This study investigated the relationship between parent-reported degree of openness and extent of problems in parent–adolescent communication and parent involvement in adolescent Type 1 diabetes management, parent and family wellbeing and adolescent glycaemic control.

**Methods:**

A cross-sectional quantitative survey was conducted. Parents completed measures of parent–adolescent communication, parent monitoring of diabetes care, diabetes family responsibility, parent knowledge of diabetes care, parent activation, parent diabetes distress, and diabetes family conflict.

**Results:**

In total, 146 parents/guardians (121 mothers, mean age 46.56 years, SD 5.18) of adolescents aged 11–17 years (mean age 13.9 years, SD 1.81) with Type 1 diabetes completed the survey. Open parent–adolescent communication was significantly correlated to adolescents’ voluntarily disclosing diabetes-specific information to their parents more frequently, increased parental knowledge of their adolescent's diabetes care completion, parents feeling more capable and willing to take action in relation to their adolescent's diabetes health, lower levels of diabetes-related parental distress, less diabetes-specific family conflict, and optimal glycaemic control.

**Discussion:**

Parent–adolescent communication has an important role to play in Type 1 diabetes healthcare management and psychosocial wellbeing during adolescence. Optimising open parent–adolescent communication represents a potentially useful target for interventional research and should be considered by healthcare professionals during healthcare encounters.

## Introduction

Type 1 diabetes mellitus constitutes a significant and growing burden in paediatric health service provision, with an increasing incidence rate of 3.4% annually in most European countries.^1^ Childhood Type 1 diabetes is advanced as a ‘family condition’, attributable to the fact that family interactions, communication styles and parents’ supervisory roles are contributors to effective childhood diabetes management and outcomes.^2^ A particularly difficult transition period for children living with Type 1 diabetes and their families is adolescence; a time where deterioration in metabolic control has been observed.^3^ While multiple factors (e.g. pubertal insulin resistance, risk-taking behaviours, poor treatment adherence) contribute to deterioration in metabolic control as adolescents mature,^4^ an additional challenge is the complexity involved in sharing diabetes management between parents and adolescents. During this developmental period, adolescents should become more autonomous, increasingly taking ownership of their diabetes management with parallel decreasing parental responsibility.^5^ However, relinquishing control of diabetes care tasks to adolescents is a notable challenge for parents.^6^ This is largely due to parental fear and worry that adolescents will not successfully negotiate their health management and encounter lasting negative consequences of suboptimal diabetes control.^7^

Sustained parental involvement and close parental monitoring of adolescents’ care completion can lead to better physical and psychosocial health outcomes for adolescents living with diabetes.^8,9^ However, parental hypervigilance or over-involvement in adolescents’ diabetes care can denote sources of stress, diabetes-related conflict and disagreement for families.^10^ Consequently, parents struggle with finding a balance between involvement and interference and knowing when to step in or step back.^6^ Assisting adolescents to assume greater responsibility for their diabetes management whilst concurrently maintaining optimal diabetes control and positive family dynamics is a challenge for parents.^6^ Psychological distress levels amongst parents of children with diabetes tend to be elevated during adolescence.^11^ Parental distress can deleteriously impact adolescent quality of life and psychological wellbeing, in addition to having negative implications for adolescent diabetes self-management, which, in turn, can further exacerbate parental distress, thereby leading to a self-perpetuating cycle where all parties are negatively affected.^11^

Parent–adolescent communication represents an important modifiable variable that, if optimised, could assist in overcoming challenges ranging from adolescents’ diabetes management to disrupted family dynamics.^12^ Dashiff et al. contended that more information was required to support parents to stabilise their involvement in diabetes management during adolescence and to nurture effective communication.^13^ Moreover, it has been postulated that the way parents and adolescents communicate could influence whether adolescents with diabetes are successful in navigating their new role in self-care.^14^ Indeed, enhancing and enabling successful familial communication is one of several targets of multi-faceted interventions yielding benefits in childhood diabetes treatment adherence and glycaemic control and in reducing diabetes-specific family conflict.^15^ A recent scoping review highlighted the importance of effective communication to clarify roles and responsibilities for diabetes management throughout adolescence.^16^

A number of variables that have been advanced as constituting specific surrogate measures and/or indicators of successful (e.g. support and warmth) or unsuccessful (e.g. conflict and control) parent–adolescent communication have previously been quantitatively examined in childhood Type 1 diabetes. Such variables have been found to correlate with adolescent glycaemic control, self-management and quality of life.^14^ A narrative review noted a shift in focus from amount to quality (i.e. warm, critical) of parent involvement in diabetes management in youths; with open communication of particular importance to facilitate high-quality parental involvement enabling efficient diabetes responsibility sharing.^9^ Conversely, low-quality (controlling, critical, restrictive) parental involvement can create family conflict and adolescents’ engagement in secretive, argumentative, or hostile behaviour can impede parents’ ability to be involved in diabetes management in a collaborative manner.^9^ However, little is known quantitatively about communication openness and extent of communication problems in families living with Type 1 diabetes during adolescence from parent perspectives. To our knowledge the relationship between openness and extent of problems in parent–adolescent communication and parent involvement in adolescent diabetes management, parent and family wellbeing, and adolescent glycaemic control has not been investigated previously. To address this gap, this study investigated the:

- Association between parent-reported degree of openness and extent of problems in parent–adolescent communication and demographic and clinical variables.
- Relationship between parent-reported degree of openness and extent of problems in parent–adolescent communication and parent involvement in adolescent Type1 Diabetes management (i.e. monitoring of diabetes care, diabetes family responsibility, parental knowledge, and activation).
- Relationship between parent-reported degree of openness and extent of problems in parent–adolescent communication and parent and family psychological wellbeing (i.e. parent distress and diabetes specific family conflict).
- Relationship between parent-reported degree of openness and extent of problems in parent–adolescent communication and adolescent glycaemic control.

## Methods

### Design, participants and recruitment

A quantitative cross-sectional survey was conducted between April and November 2018. Eligibility criteria were parents/caregivers (i.e. biological parents and/or legal guardians) of adolescents aged 11–17 years with a diagnosis of Type 1 diabetes for 6 months or longer. With the exception of hyperthyroidism, hypothyroidism and coeliac disease (co-morbidities commonly associated with Type 1 diabetes), parents were ineligible to participate if their adolescent with diabetes had (or had previously experienced) any other significant chronic medical condition(s) and/or had notable cognitive impairment or developmental delay.

Ethical approval was granted by participating Hospital and University Research Ethics Committees (TSCUH/16.056 and DCUREC/2016/144). Cross-sectional surveys were designed to assess the relationship between parents’ perceptions of degree of openness and extent of problems in parent–adolescent communication and: (1) demographic and clinical variables; (2) parental involvement in adolescents’ diabetes management; (3) parent and family psychological wellbeing; and (4) adolescent glycaemic control. Surveys were available for completion in hard-copy format or online. Return/online submission of the survey signified parental consent.

Eligible parents/caregivers were recruited through one of three routes. (i) A nominated gatekeeper at two Diabetes and Endocrine Clinics at a national paediatric hospital identified eligible parent participants from adolescent outpatient clinic lists. A study pack (i.e. invitation letter, plain language statement, survey questionnaire and return envelope) was distributed by post to potential participants. (ii) Parents of adolescents who had agreed to follow-up contact to consider taking part in the quantitative survey, following participation in an earlier qualitative study, were contacted and a study pack was posted to interested participants. (iii) Further parent participants were recruited through open advertisements placed on website and social media pages of the national diabetes association where parents could access study information and complete the survey online or request a hard copy version.

### Measures

The survey questionnaire included a number of empirically validated measures to investigate whether there were correlations between parent-reported degree of openness and extent of problems in parent–adolescent communication as measured by the parent version of the parent–adolescent communication scale (PACS)^17^; and (i) parent involvement in adolescent diabetes management as measured by revised version of the parent monitoring of diabetes care questionnaire (PMDC-R),^18^ parent version of the diabetes family responsibility questionnaire (DFRQ),^19^ parent knowledge of diabetes care questionnaire (PKDC),^18^ and parent patient-activation measure (PPAM-13)^20^; and (ii) parent and family psychological wellbeing as measured by parent diabetes distress scale (DDS),^21^ and parent version of the revised version of the diabetes family conflict scale (DFCS-R).^22^ See Table 1 for an overview of measures including internal consistency values for each scale/subscale. In the current sample, Cronbach's alpha for ‘healthcare team distress’ subscale of DDS was below the recommended value of 0.7 and this subscale was excluded from analysis.

**Table 1.** Overview of survey measures.

Construct	Measure	Description	No. of items	Scale	Scores	Cronbach's alpha for measures in this study
Parent–adolescent communication (parent-reported)	Parent version of the parent–adolescent communication scale (PACS)	Two subscales assessing specific domains of generic parent–adolescent communication 1. degree of openness (10 items) 2. extent of problems (10 items)	20	Items rated from 1 to 5 on a 5-point Likert-type scale	Higher scores for each subscale indicative of more open communication and fewer problems with communication Total scores for subscales were computed and used for statistical analysis	*α* = 0.78 (degree of openness subscale) *α* = 0.80 (extent of problems subscale)
Parent involvement	Revised version of the parent monitoring of diabetes care questionnaire (PMDC-R)	Scale examines parent supervision and monitoring of adolescent diabetes care behaviours 1. parents’ direct observation and presence (DOP) 2. parents soliciting information from youths (SIY) 3. youths disclosing (YD) care-related information to parents	27	Items scored on a 5-point response scale ranging from 1 to 5	Higher scores reflect higher levels of parent monitoring A total scale score can be computed, as well as three subscale scores that assess the domains of monitoring	*α* = 0.89 (total scale) *α* = 0.83 (DOP subscale) *α* = 0.82 (SIY subscale) *α* = 0.85 (YD subscale)
	Parent version of the diabetes family responsibility questionnaire (DFRQ)	Assesses family members’ perceptions of whom within the family assumes responsibility for a broad range of specific tasks (i.e. regimen tasks, general health monitoring and social presentation of diabetes) related to managing the child's diabetes	17	Items rated on a 3-point response scale ranging from 1 to 3	Score of 1 reflects the child initiating responsibility for diabetes-specific tasks the majority of the time Score of 2 represents parent and child sharing responsibility Score of 3 indicates parents initiating responsibility for diabetes-specific tasks most of the time	*α* = 0.81
	Parent knowledge of diabetes care questionnaire (PKDC)	Measures parents’ knowledge of whether or not their youth has completed their diabetes care	14	Items rated on a 5-point Likert-type scale (scores range from 1 to 5)	Higher scores reflect a higher level of parental knowledge of their youths’ engagement with diabetes care	*α* = 0.91
	Parent patient-activation measure (PPAM-13)	Assesses parents’ competence and readiness to take action concerning their child's health condition and advocate on their child's behalf	13	Items rated on a 5-point response scale	Higher scores reflect higher levels of parent activation A N/A response is assigned a value of 0 Scores where items are applicable to parents are assigned values ranging from 1 to 4	*α* = 0.91
Parent and family Wellbeing	Parent diabetes distress scale (DDS)	Measures emotional burdens and worries of parents of adolescents with Type 1 diabetes overall and in four specific domains 1. personal distress 2. teen management distress 3. parent/teen relationship distress 4. healthcare team distress	20	Items rated on a 5-point Likert-type scale; ratings assigned values from 0 to 4	Higher scores represent greater levels of parental distress related to their youths’ diabetes	*α* = 0.94 (total scale) *α* = 0.83 (personal distress subscale) *α* = 0.79 (teen management distress subscale) *α* = 0.94 (parent/teen relationship distress subscale) *α* = 0.57 (healthcare team distress subscale)
	Parent version of the revised version of the diabetes family conflict scale (DFCS-R)	Measures the extent of diabetes-specific conflict within families related to direct and indirect diabetes management tasks (e.g. blood glucose monitoring, insulin administration and disclosing the diagnosis to others)	19	Items rated on 3-point Likert-type scale with scores ranging from 1 to 3	Higher scores indicative of greater levels of diabetes-specific family conflict A total scale score is computed for this scale	*α* = 0.87

Parent demographic data included age, gender, education level and relationship to adolescent. Adolescent demographic and clinical variables included age, gender, time since diagnosis, family history of diabetes, blood glucose monitoring technique, insulin administration modality and most recent glycated haemoglobin (HbA1c). For HbA1c, parents were asked to report their adolescent's most recent outpatient clinic HbA1c result. For this item, parents could select one of eight possible responses ranging from less than 6.5% (i.e. <48 mmol/mol) to more than 9.5% (i.e. >80 mmol/mol). An ‘I don’t know’ option was also available for parents who could not recall their adolescent's most recent HbA1c value (these values were excluded from analyses).

### Statistical analysis

Statistical analyses were performed using IBM Statistics Package for Social Sciences, Version 24.0. Data were cleaned and coded, and mean and total scores for scales and subscales computed. Correlational and group difference analyses were performed to investigate the relationship between parent-reported degree of openness and extent of problems in parent–adolescent communication and parent involvement in adolescent diabetes management, parent and family psychological wellbeing and demographic and clinical variables. The decision as to whether specific data points merited use of parametric or non-parametric correlational and group difference analyses was made a priori based on examination of skewness and kurtosis values of data, as well as Shapiro–Wilks Normality test *p* values. Parent-reported PACS data were non-normally distributed (*p *< .05) and it was deemed prudent to conduct non-parametric statistical analyses (i.e. Spearman's rho correlational analyses and Mann–Whitney *U* and Kruskal–Wallis *H* group difference analyses). For glycaemic control analysis, raw HbA1c results parents provided were used, but, additionally, results were recoded into two categories, namely ≤7.0%, that is <53 mmol/mol and >7.0%, that is >53 mmol/mol HbA1c, in line with the International Society for Pediatric and Adolescent Diabetes recommendations^23^ where ≤7.0% is considered optimal HbA1c, for further analysis.

## Results

A total of 146 parents/guardians (121 mothers, mean age 46.56 years, SD 5.18) of adolescents aged 11–17 years (mean age 13.9, SD 1.81) with a diagnosis of Type 1 diabetes for 6 months or longer participated. Demographic characteristics of parent participants, and demographic and clinical profile of the adolescents with diabetes, are summarised in Table 2. Descriptive statistics for each outcome measure and subscales are presented in Table 3.

**Table 2.** Demographic and clinical characteristics of parent participants and their adolescent children.

Variable	Total *n* (%)	Mean (SD) [Range]
Parent participants (*n* = 146)		
*Age (years)*		46.56 (5.18) [33–59]
*Gender* Male Female Did not complete item	24 (16.4%) 121 (82.9%) 1 (0.7%)	
*Education level* Junior certificate Leaving certificate FETAC^a^ award Primary degree Postgraduate degree Other ^b^ Did not complete item	6 (4.1%) 41 (28.1%) 25 (17.1%) 29 (19.9%) 28 (19.2%) 10 (6.8%) 7 (4.8%)	
*Relationship to adolescent* Mother Father Legal guardian	122 (83.6%) 23 (15.8%) 1 (0.7%)	
Adolescent children of parent participants		
*Age (years)*		13.89 (1.81) [11–17]
*Time since Type 1 diabetes diagnosis (years)*		5.76 (3.48) [<1–15]
*Gender* Male Female	79 (54.1%) 67 (45.9%)	
*Family history of diabetes (parents, siblings or grandparents)* Yes No	29 (19.9%) 117 (80.1%)	
*Blood glucose monitoring technique* Finger prick test only Flash glucose monitoring system ^c^ Continuous glucose monitoring device ^c^ Other method	46 (31.5%) 82 (56.2%) 16 (11.0%) 2 (1.4%)	
*Insulin administration modality* Multiple daily injections ^d^ Insulin pump	51 (34.9%) 95 (65.1%)	
*Most recent HbA1c result* ≤6.5% (≤48 mmol/mol) 6.6–7.0% (49–53 mmol/mol) 7.1–7.5% (54–58 mmol/mol) 7.6–8.0% (60–64 mmol/mol) 8.1–8.5% (65–69 mmol/mol) 8.6–9.0% (70–75 mmol/mol) 9.1–9.5% (76–80 mmol/mol) >9.5% (>80 mmol/mol) I don’t know Did not complete item	13 (8.9%) 21 (14.4%) 23 (15.8%) 34 (23.3%) 28 (19.2%) 7 (4.8%) 3 (2.1%) 7 (4.8%) 6 (4.1%) 4 (2.7%)	

a Further Education and Training Awards Council.

b Education listed under ‘Other’ by participants included post-graduate diplomas, as well as technical and professional examinations.

c Plus finger prick test.

d Using an insulin pen or syringe.

**Table 3.** Mean scores and standard deviations for questionnaire measures and subscales.

Measure	Mean	SD
Parent version of the parent–adolescent communication scale (PACS)	77.43	12.0
Degree of openness	39.90	5.90
Extent of problems	37.53	7.48
Revised version of the parent monitoring of diabetes care questionnaire (PMDC-R)	3.35	0.68
Parents’ direct observation and presence (DOP)	4.11	0.75
Parents soliciting information from youths (SIY)	4.28	0.94
Youths disclosing (YD) care-related information to parents	2.94	1.05
Parent version of the diabetes family responsibility questionnaire (DFRQ)	35.59	4.90
Parent knowledge of diabetes care questionnaire (PKDC)	4.43	0.43
Parent patient-activation measure (PPAM-13)	72.56	17.69
Parent diabetes distress scale (DDS)	1.31	0.86
Personal distress	1.32	0.99
Teen management distress	2.11	1.09
Parent/teen relationship distress	1.13	1.05
Healthcare team distress	0.42	0.74
Parent version of the revised version of the diabetes family conflict scale (DFCS-R)	25.92	5.53

### Parent–adolescent communication and parent demographic variables

For parent–adolescent communication, neither parent-reported degree of openness or extent of problems were significantly related to parental age (Table 4); nor were significant group differences revealed for parent–adolescent communication variables according to parental gender or education level (Table 4).

**Table 4.** Correlations and group differences for parent–adolescent communication (parent-reported) and demographic and clinical variables, and glycaemic control.

Variable	Parent–adolescent communication: openness subscale (*ρ* or *U* or *X*^2^)	Parent–adolescent communication: extent of problems subscale (*ρ* or *U* or *X*^2^)
Parent–adolescent demographics		
Parent age	-0.012 (*ρ*)	-0.010 (*ρ*)
Adolescent age	-0.124 (*ρ*)	-0.074 (*ρ*)
Parent gender	1740.5 (*U*)	1811.5 (*U*)
Adolescent gender	3022.5 (*U*)	2855.5 (*U*)
Parent education level	2.76 (*X*^2^)	8.68 (*X*^2^)
Adolescent clinical variables		
Time since Type 1 Diabetes diagnosis	-0.064 (*ρ*)	-0.030 (*ρ*)
Family history of diabetes	1666.5 (*U*)	1790.0 (*U*)
Blood glucose monitoring technique	5.22 (*X*^2^)	.502 (*X*^2^)
Insulin administration modality	2468.0 (*U*)	2594.0 (*U*)
Glycaemic control		
Most recent HbA1c result (8 categories)	19.04 (*X*^2^)**	12.48 (*X*^2^)
Most recent HbA1c result (ISPAD categories)	1171.5 (*U*)**	1294.0 (*U*)*

*P < 0.05.

**P < 0.01.

### Parent–adolescent communication and adolescent demographic and clinical variables

Neither parent-reported degree of openness nor the extent of problems subscales of parent–adolescent communication were significantly related to adolescent age or duration of time since adolescent diabetes diagnosis (Table 4). Additionally, no group differences were identified for either parent–adolescent communication subscale according to adolescent gender, family history of diabetes, blood glucose monitoring technique or insulin administration modality (Table 4).

### Parent–adolescent communication and parent involvement in adolescent diabetes management

More open parent–adolescent communication and fewer problems in parent–adolescent communication were each significantly related to higher levels of youth disclosure of diabetes-related issues to their parents, greater parental knowledge of their adolescents’ completion of diabetes care, and increased parental activation with respect to the adolescent's health condition (Table 5).

**Table 5.** Correlations between parent–adolescent communication (parent-reported) and parental involvement in diabetes management and parent and family wellbeing variables.

Variable	Parent–adolescent communication: openness subscale (*ρ*)	Parent–adolescent communication: extent of problems subscale (*ρ*)
Parent involvement in adolescent diabetes management		
*Parent monitoring of diabetes care-revised scale*		
Total scale	-0.076	0.119
Direct observation and presence subscale	-0.018	-0.066
Soliciting information from youths subscale	-0.056	-0.129
Youth disclosure subscale	0.265**	0.353***
*Diabetes family responsibility questionnaire*	-0.138	-0.139
*Parent knowledge of diabetes care questionnaire*	0.196*	0.255**
*Parent patient activation measure*	0.297***	0.252**
Parent and family psychological wellbeing		
*Parent diabetes-related distress scale*		
Total scale	-0.377***	-0.469***
Personal distress subscale	-0.267**	-0.354***
Teen management distress subscale	-0.205*	-0.188*
Parent–teen relationship distress subscale	-0.455***	-0.610***
*Diabetes-specific family conflict scale*	-0.379***	-0.490***

*P < 0.05.

**P < 0.01.

***P < 0.001.

Neither degree of openness nor extent of problems in parent–adolescent communication were significantly related to parental monitoring of their adolescents’ diabetes care overall, directly observing and/or being present during adolescent care tasks, soliciting information from youths, or parent perspectives on whom within the family assumes responsibility for adolescents’ diabetes care (Table 5).

### Parent–adolescent communication and parent and family psychological wellbeing

More open parent–adolescent communication and fewer problems as reported by parents in terms of parent–adolescent communication were each significantly correlated with parents reporting less diabetes-related distress overall, less personal diabetes-related distress, less distress related to managing their adolescent with diabetes, less distress with regard to the adolescent-parent relationship, and less diabetes-related family conflict (Table 5).

### Parent–adolescent communication and adolescents’ glycaemic control

Significant group differences were identified in parent–adolescent communication according to adolescent glycaemic control. When adolescents’ most recent HbA1c results were categorised into ≤7.0% and >7.0% groups, parents of adolescents with optimal HbA1c results (≤7.0%) reported significantly higher levels of openness and significantly fewer problems in relation to communicating with their adolescent with diabetes (Table 4).

## Discussion

This study has shown that parent-reported openness in parent–adolescent communication is significantly associated with: (1) greater disclosure of diabetes-pertinent information by adolescents to their parents; (2) enhanced parental knowledge of their adolescents’ diabetes care completion; (3) increased parental confidence and competence in taking action in relation to their adolescent's diabetes; (4) reduced parental diabetes-related distress; (5) lower levels of diabetes-specific family conflict; and (6) improvement in adolescent glycaemic control. We also found that more problems in parent–adolescent communication was significantly related to: (1) adolescents voluntarily disclosing less information about their diabetes care to their parents; (2) parents being less knowledgeable about adolescent diabetes care completion; (3) parents perceiving themselves as being less capable and less likely to advocate on behalf of their child's health condition; (4) increased diabetes-specific parental distress; (5) greater diabetes-specific family conflict; and (6) sub-optimal adolescent glycaemic control.

Voluntary disclosure of healthcare-related information to parents by adolescents represents a positive adaptive coping strategy indicative of a collaborative and consultative approach to diabetes management; an approach that has long been endorsed and favoured during adolescence for easing transition and shift towards more autonomous health management.^24^ Parents’ awareness of adolescent diabetes care completion is important because it is not only associated with positive healthcare outcomes, but also with reduced parental distress which is suggested to mediate the relationship between parental knowledge and better glycaemic control.^25^ The links between more open parent–adolescent communication and higher levels of adolescent disclosure (parent-reported), and greater parental knowledge of children's diabetes care completion, identified in our study, highlights the importance of parents maintaining open communication channels to assist adolescents with diabetes to navigate this complex transition period in their lives. On the contrary, problematic parent–adolescent communication appears to foster maladaptive healthcare coping behaviours. These findings support previous research from adolescent's perspectives where open communication was found to facilitate diabetes responsibility sharing, with higher adolescent disclosure to parents about diabetes management associated with improved adolescent adherence to self-management responsibilities, whereas greater adolescent secrecy was associated with poorer adherence.^26^

The significant correlation between parent–adolescent communication and parents’ self-belief in their ability and willingness to act on behalf of their adolescent's health condition is noteworthy. This could be partially attributable to the fact that less parental confidence and advocacy surrounding their adolescent's diabetes could act as a stimulus for more problems in parent–adolescent communication: with adolescents perceiving such actions as parental disinterest in their care and/or their parents’ inability to provide them with adequate support.

Parental distress in families living with childhood Type 1 diabetes extends beyond impacting parents alone. Parental distress is additionally deleteriously linked to adolescents’ health^27^ and psychological wellbeing.^11^ The link between open parent–adolescent communication and lower diabetes-related parental distress (across three distress domains) identified in our study suggests that encouraging and supporting openness between parents and adolescents with diabetes, by identifying and eliminating barriers to open communication, could improve parents’ psychological wellbeing whilst concurrently positively influencing family dynamics and adolescent health status.

Evidence from a systematic review revealed that irrespective of source, high levels of intra-familial conflict are associated with detrimental effects on both self-care and metabolic control in youths with Type 1 diabetes.^28^ Our finding that more problems in parent–adolescent communication correlates with higher levels of diabetes-specific familial conflict has implications for adolescent health outcomes and how adolescents engage with their health care. In our study, fewer problems in parent–adolescent communication were significantly related to improved glycaemic control. This highlights the importance of enhancing parent–adolescent communication in families living with childhood Type 1 diabetes to improve both clinical and psychosocial outcomes.

Our findings have a number of implications for healthcare professionals and diabetes support organisations. Given that positive outcomes are associated with more openness and fewer problems in parent–adolescent communication, family communication should be considered by healthcare professionals during consultations. Healthcare professionals have an important role to play in observing for, attending to cues of and recognising the manifestations of problematic communication amongst parents and adolescents living with Type 1 diabetes. Assisting families to adopt open communicative strategies and to avail of appropriate resources, interventional programmes and/or support agencies, and having access to psychological expertise in the multi-disciplinary team, could result in positive outcomes for adolescent health management and parent and family psychological wellbeing.

There are some limitations to the current study. First, self-selecting parent participants were under-representative of fathers. Future studies may wish to specifically recruit fathers to investigate their perspectives on parent–adolescent communication, acknowledging that previous evidence has highlighted the important role fathers play in influencing childhood diabetes outcomes.^29^ Second, although there is no diabetes childhood national register against which we can compare our sample, parental mean age is high (i.e. average parent/guardian was 32 years older than the adolescent). Anecdotal evidence from clinics suggests that parental age might be a proxy for social advantage; therefore, future studies should target younger parents. Third, HbA1c values were self-reported by parents rather than obtained from medical records. Reliance on parental recall may have yielded erroneous values in some instances. Parents were only asked to report their adolescent's HbA1c results based on their most recent healthcare consultation. This surrogate marker is only indicative of adolescents’ glycaemic control for the previous 3 months. Finally, our study was cross-sectional, therefore causal direction of relationships cannot be determined.

This study addresses a limitation identified in existing literature on childhood Type 1 diabetes during adolescence by exploring parent perspectives of the role of communication (openness and extent of problems) on health management behaviours and psychological outcomes.^13,14^ Future research should explore adolescent and parent–adolescent dyadic perspectives to identify whether variations exist in the role openness or extent of problems in parent–adolescent communication plays in influencing how each party adjusts to changes in health management behaviour during adolescence; and any consequences for adolescent health outcomes and family psychological wellbeing. Further longitudinal investigation is needed to assess long-term effects of optimising effective parent–adolescent communication in families living with Type 1 diabetes in adolescence.

## Conclusion

Parent–adolescent communication plays an important role in childhood Type 1 diabetes management and parent and family wellbeing during adolescence. Open communication and fewer problems in parent–adolescent communication was associated with positive outcomes. Enhancing open parent–adolescent communication and reducing problems in parent–adolescent communication presents a potential avenue for interventional research in families living with Type 1 diabetes in adolescence.
